# Predictors for delayed encephalopathy following acute carbon monoxide poisoning

**DOI:** 10.1186/1471-227X-14-3

**Published:** 2014-01-31

**Authors:** Kaoru Kudo, Kotaro Otsuka, Junko Yagi, Katsumi Sanjo, Noritaka Koizumi, Atsuhiko Koeda, Miki Yokota Umetsu, Yasuhito Yoshioka, Ayumi Mizugai, Toshinari Mita, Yu Shiga, Fumito Koizumi, Hikaru Nakamura, Akio Sakai

**Affiliations:** 1 Department of Neuropsychiatry, Iwate Medical University, school of Medicine, Morioka, Iwate, Japan; 2 Department of Disaster & Community Psychiatry, Iwate Medical University, school of Medicine, Morioka, Iwate, Japan; 3 Department of Critical Care Medicine, Iwate Medical University, school of Medicine, Morioka, Iwate, Japan

**Keywords:** Delayed encephalopathy, Carbon monoxide poisoning, Delayed neuropsychiatric sequelae, Suicide attempt, Psychiatric emergency

## Abstract

**Background:**

In Japan, many carbon monoxide (CO) poisoning cases are transported to emergency settings, making treatment and prognostic assessment an urgent task. However, there is currently no reliable means to predict whether “delayed neuropsychiatric sequelae (DNS)” will develop after acute CO poisoning. This study is intended to find out risk factors for the development of DNS and to characterize the clinical course following the development of DNS in acute CO poisoning cases.

**Methods:**

This is a retrospective cohort study of 79 consecutive patients treated at a single institution for CO poisoning. This study included 79 cases of acute CO poisoning admitted to our emergency department after attempted suicide, who were divided into two groups consisting of 13 cases who developed DNS and 66 cases who did not. The two groups were compared and analyzed in terms of clinical symptoms, laboratory findings, etc.

**Results:**

Predictors for the development of DNS following acute CO poisoning included: serious consciousness disturbance at emergency admission; head CT findings indicating hypoxic encephalopathy; hematology findings including high creatine kinase, creatine kinase-MB and lactate dehydrogenase levels; and low Global Assessment Scale scores. The clinical course of the DNS-developing cases was characterized by prolonged hospital stay and a larger number of hyperbaric oxygen (HBO) therapy sessions.

**Conclusion:**

In patients with the characteristics identified in this study, administration of HBO therapy should be proactively considered after informing their family, at initial stage, of the risk of developing DNS, and at least 5 weeks’ follow-up to watch for the development of DNS is considered necessary.

## Background

Carbon monoxide (CO) poisoning has been a preferred method of suicide due to its high success rate of approximately 30% [1], its simplicity, and the minimal external injury involved. According to the 2011 “White Paper on Suicide (*Jisatsu Taisaku Hakusho*)” compiled by the Japanese Cabinet Office, “briquettes or similar materials” were the second most used means of suicide used by men (2,137 persons, 10.2%), next only to “hanging” (14,354 persons, 68.5%), with their use spread across a wide range of age groups from those in their twenties to those in their sixties [2].

In general, CO poisoning occurs due to such causes as inhalation of exhaust gas from automobiles, or incomplete combustion of charcoal, briquettes, fuel gas or oil in a closed place, or in such settings as a fire. Its pathology consists mainly of dysfunction of various organs due to tissue hypoxia. Since hypoxia is reversible, early removal of CO is essential and high levels of oxygen should be administered wherever possible during transportation, examination and treatment. Means to administer high concentrations of oxygen include normobaric oxygen (NBO) and hyperbaric oxygen (HBO) therapies. Previous studies comparing the two therapies have reported that HBO therapy is effective as a treatment to reduce the incidence of DNS and reduce its severity in cases of acute CO poisoning [3-6]. Other studies, however, have disputed that finding, so there is still worldwide controversy regarding the effectiveness of HBO therapy [7]. In addition, various studies worldwide have cited different criteria for administering HBO therapy during acute CO poisoning due to the ambiguity of indices of the clinical severity of acute CO poisoning. Criteria for administering HBO therapy have yet to be standardized. Moreover, a patient transfer from a medical facility with no HBO chamber to a facility with an HBO chamber has to be considered [8].

CO poisoning is generally classified as acute CO poisoning or chronic CO poisoning depending on the duration of CO exposure. CO poisoning is categorized into different forms based on the clinical manifestations resulting from CO exposure over time. With acute CO poisoning, the patient recovers without sequelae, but with delayed CO poisoning or intermittent CO poisoning the patient can be left with neurologic sequelae.

Delayed CO poisoning refers to impaired consciousness that develops at the time of poisoning and that persists without improving. This form of poisoning causes brain cells to be deprived of oxygen and can lead to sequelae such as amnestic syndrome, loss of initiative, affective incontinence, and parkinsonism [9]. Intermittent CO poisoning is pathology where, after a certain asymptomatic period (from several days to four weeks; an average of two weeks) following recovery from acute-phase symptoms, neuropsychiatric symptoms develop rapidly, such as amnesia, disorientation, loss of mathematical ability, slowness of movement, urinary incontinence, apathy, anxiety or emotional lability. These symptoms may lead to Apallic syndrome in some cases and/or to death in worst cases [5]. Intermittent CO poisoning is thought to develop as a result of the progression of focal demyelination of the cerebral white matter and subsequent neuronal death. Intermittent CO poisoning has been reported to occur in 2.8% of acute CO poisoning cases and 11.8% of those who were hospitalized [10]. These two types of CO poisoning are sometimes referred to collectively as “delayed neuropsychiatric sequelae (DNS).”

Iwate Medical University Hospital (“Hospital”) has an HBO chamber, and its emergency department accepts more than 10 cases of attempted suicide with CO poisoning annually. The Hospital sometimes accepts CO poisoning cases in a very acute phase from neighboring medical institutions with no HBO chamber. Virtually all of these cases have been hospitalized after admission and have received treatment including HBO therapy, with some developing DNS and remaining having been hospitalized for prolonged periods.

While it is necessary to predict the potential development of DNS at the initial stage following admission to the emergency department, no correlation has been found between CO-Hb level in the blood and clinical severity [11]. It has also been found impossible to predict prognosis from EEG findings obtained at the initial stage [12]. Based on the fact that DNS is caused by demyelinating changes in the cerebral white matter, some researchers have pointed out the need to measure myelin basic protein (MBP) levels in the cerebrospinal fluid (CSF) soon after injury [13], as well as to assess nerve fibers in the white matter by diffusion tensor imaging [14] or 1H-magnetic resonance spectroscopy [15]. However, since head MRI depicts all the various histological changes, it may not be possible to accurately tell the progress of the condition in the cerebral white matter [16]. In addition, there have been cases who developed DNS despite having subnormal MBP levels in the CSF two weeks immediately following injury [17]. A recent study examined development of cognitive sequelae and genetic factors 6 weeks after CO poisoning. The study found that the apolipoprotein (APOE) epsilon4 allele was not associated with development of cognitive sequelae [18]. Taken together, at present no reliable means to predict DNS have been established, making prediction during the acute phase difficult.

However, if the development of DNS can be predicted during the acute phase, it would help making decisions on treatment strategy, by such means as identifying cases to which HBO therapy should be actively administered and setting an appropriate period of hospital treatment. In the present study, we studied cases of attempted suicide with acute CO poisoning admitted to our emergency department, and reviewed and analyzed these cases with the intention of identifying risk factors for developing DNS and characterizing the clinical course after the development of DNS.

## Methods

This is a retrospective cohort study of 79 consecutive patients treated at a single institution for CO poisoning. This study included 79 cases who were admitted to the Hospital’s emergency department due to CO poisoning after attempted suicide during the period between 2002 and 2011. All subjects were divided into two groups, consisting of 13 cases who developed DNS and 66 cases who did not, and were reviewed for clinical symptoms and laboratory findings at admission to the emergency department, circumstances of injury, treatment received during the acute phase, and other information to the extent available in the emergency department setting.

Patient medical records were retrospectively reviewed, and patients who developed DNS and patients who did not develop DNS were compared in terms of 16 items: gender, age, location of exposure, estimated duration of exposure, whether or not the patient was transported from another hospital, severity of impaired consciousness (i.e., Japan Coma Scale [JCS] score) when the patient was first seen at a hospital [19], CO-Hb level when the patient was first seen at a hospital, white blood cell (WBC) count and CK, CK-MB, and LDH levels on the day the patient was seen, whether or not there were abnormal findings from a head CT scan when the patient was first seen, whether or not HBO therapy was administered on the day the patient was seen, diagnostic category according to “Mental and behavioural disorders” in the International Classification of Diseases, Tenth Revision (ICD-10) [20], duration of hospital stay, and number of sessions of HBO therapy.

In addition, the patients were assessed in terms of their psychiatric symptoms using a Japanese version (by Kitamura, et al.) [21] of the Oxford University version of the Brief Psychiatric Rating Scale (BPRS), and were also investigated in terms of their general psychiatric symptoms and abilities of daily living using a Japanese translation by Kitamura, et al. of the Global Assessment Scale (GAS) [22]. Furthermore, the patients’ life events prior to their attempted suicide were assessed using the Life Change Units (LCU) of the Holmes Social Readjustment Rating Scale [23].

Note that JCS scores and CO-Hb levels used in this study were those obtained at the first medical institution, not necessarily the Hospital, to which each patient was admitted in emergency, since quite a few cases were transferred to the Hospital under oxygen administration after consultation at another medical institution.

Assessment and diagnosis of each review item were performed by an emergency psychiatrist or the Hospital’s psychiatrist on duty.

Statistical processing was performed using SPSS 17.0J for Windows. Testing of mean values, ratios and JCS scores was conducted using one-way analysis of variance, a chi-square test and the Mann-Whitney U test, respectively. In all tests, the level of significance was 5%, with significance probabilities being expressed in numbers.

This study is a chart review study and we did not obtain informed consent. Personally identifiable information was excluded from data. Consideration was given to the protection of personal information in the process of data management and processing. This study was conducted with the approval of the Ethics Committee of the Iwate Medical University, School of Medicine.

## Results

The mean age of all 79 cases was 40.44 years (men, 43.0 ± 14.23 years; women, 35.0 ± 12.57 years). Nineteen cases (24%) were transferred to the Hospital from their previous respective medical institutions to which they were first brought, on the grounds of difficulty providing care. The 13 cases who developed DNS consisted of 11 men and 2 women and had a mean age of 47.38 ± 14.83 years, two of who had been transferred from another hospital. In terms of type of DNS, 5 cases had intermittent CO poisoning, whereas the remaining 8 cases included cases of prolonged CO poisoning and those of persistent apallic syndrome (see the Table 1). The mean period before the onset of intermittent CO poisoning was 23.2 days of illness.

**Table 1:** Comparison of characteristics between the delayed neuropsychiatric sequelae (DNS)-developing group and the non-DNS-developing group

**Item**	**Category**	**DNS-developing group**	**Non-DNS-developing group**	**P value**
		**n = 13**	**n = 66**	
Gender	Male (%)	11 (84.6)	54 (81 8)	1.000
	Female (%)	2 (15.4)	12 (18.2)	
Mean age ± S.D. (years)		47.38 ± 1483	40 44 ± 1391	108.000
Place of exposure	Car (%)	10 (76.9)	47 (71.2)	1.000
	Room (%)	3 (23.1)	19 (28.8)	
The number of the transfered from outside institution	(%)	2 (15.4)	17(25.8)	0.723
Time to onset of intermittent CO poisoning (day)		23.2		
Mean duration of exposure ± SD (min)		310.00 ± 242.49	253.33 ± 199.93	0.644
Mean JCS score at first hospital consultation ± S.D.		200.4 ± 107.24	52.71 ± 94.55	0.000*
Abnormal head CT findings at consultation	Yes (%)	10 (76.9)	4 (6.2)	0.000^#^
	No (%)	3 (23.1)	61 (93.8)	
CO-Hb level at first hospital consultation ± S.D. (%)		19.58 ± 20.03	24.99 ± 13.98	2.242
Mean WBC count ± S.D. (/μ)		14391.52 ± 4534.60	13834.44 ± 10769.56	0.856
Mean CK level ± S.D. (IU/L)		4371.92 ± 5976.33	650.86 ± 2384.67	0.001I^†^
Mean CK-MD level ± S.D. (IU/L)		51.22 ± 33.28	15.66 ± 23.47	0.000^†^
Mean LDH level ± S.D. (U/L)		395.80 ± 270.04	221.87 ± 101.95	0.000^†^
HBO therapy administered at consultation?	Yes	10 (76.9)	39 (59.1)	0.350
	No	3 (23.1)	27 (40.9)	
Total BPRS score ± S.D.		14.31 ± 11.86	14.58 ± 9.124	0.925
GAS ± S.D.		17.31 ± 18.50	28.48 ± 16.70	0.033^†^
LCU ± S.D.		50.40 ± 15.05	65.15 ± 53.23	0.392
ICD-10 classification	F1 (%)	0 (0)	1 (1.5)	0.804
	F2 (%)	0 (0)	6 (9.1)	
	F3 (%)	9 (69.2)	42 (63.6)	
	F4 (%)	2 (15.4)	12 (18.2)	
	F6 (%)	1 (7.7)	3 (4.5)	
	Others (%)	1 (7.7)	2 (3.0)	
Mean length of hospital stay ± S.D. (days)		251.08 ± 283.27	37.18 ± 37.93	0.000^†^
Mean number of HBO sessions ± S.D. (sessions)		51.62 ± 16.69	5.38 ± 7.70	0.000^†^

*Total number of cases may be different for different items.

1) ^#^Chi-square test.

2) ^†^ANOVA (one way analysis of variance).

3) ^*^Mann-Whitney test.

### Patients’ background and circumstances

While the mean age was higher in the DNS-developing group by approximately 7 years, there was no significant difference between the two groups. Although approximately 80% of all cases were men, there was no significant difference in the development of DNS between male and female cases. Place of exposure to CO was broadly classified into car and room, with no significant difference between the DNS-developing and non-DNS-developing groups. Estimated duration of exposure was unknown for approximately 50% of all cases, with no significant difference between the DNS-developing and non-DNS-developing groups.

### Physical findings and laboratory results at first consultation

The patients in the DNS-developing group had significantly more severe consciousness disturbance (in terms of mean JCS score) at the time of first hospital consultation (p<0.001). A significantly higher proportion of these patients showed abnormal head CT findings indicating hypoxic encephalopathy (p<0.001). Hematology results showed that these patients also had significantly higher CK, CK-MB and LDH levels (p = 0.001, p<0.001 and p<0.001, respectively). The GAS scores of these patients, which assess their psychiatric symptoms, tended to be significantly lower than in the non-DNS-developing group (p = 0.033).

Overall, F3 was the single most common main diagnosis according to ICD-10, followed by F4, which was a tendency also shared by both groups. Severity of psychiatric symptoms (BPRS score) and life events (mean LCU score) showed no significant difference between the groups.

CO-Hb levels at the time of first hospital consultation were higher in the non-DNS-developing group, whereas WBC count was higher in the DNS-developing group, with neither showing a significant difference.

### Clinical course after hospitalization

Of the items to assess the clinical course after hospitalization, length of hospital stay (p<0.001) and the number of HBO therapy sessions (p<0.001) showed a significant difference between the two groups, with the DNS-developing group having longer hospital stay and a larger number of sessions. In the DNS-developing group, the period of time before the onset of intermittent CO poisoning ranged from 17 to 35 days (mean: 23.2 days of illness). Whether or not HBO therapy was administered immediately after consultation was not significantly different between the two groups.

## Discussion

### Patients’ background and circumstances

It has been reported that aging promotes DNS as a complication of CO poisoning [8], and that no DNS-developing cases were seen in patients younger than 30 years of age [10]. Based on these reports, we initially expected that increased general fragility caused by aging may lead to the development of DNS. However, in the present study the youngest DNS case was 23 years old and, although the mean age tended to be higher in the DNS-developing group, there was no significant difference in mean age between the DNS-developing and non-DNS-developing groups.

The location of exposure was generally classified as in a room at home and in a car. Pavese, et al. and O’Donnell, et al. state that CO gas concentration at the scene multiplied by duration of exposure is an important determinant of the severity of acute CO poisoning [24,25]. While CO gas concentration is likely to be affected by the size of the space and the time to filling the space with CO, no significant difference was observed in place of exposure or estimated duration of exposure.

Results regarding estimated duration of exposure may be affected by the fact that duration of exposure was known only for half of all cases. Specific circumstances of exposure were varied, such as a case who had prolonged exposure to CO by burning briquettes in a car and frequently getting in and out of the car to vomit outside, and another who had prolonged exposure in a well-ventilated wooden shed. While the product of CO gas concentration and duration of exposure cannot properly be calculated without collecting detailed information on individual circumstances, there is a limit to information available for collection in acute clinical settings. It is therefore considered that the wide variety of circumstantial factors involved in space and duration of exposure prevented any significant difference in these factors from being detected with respect to the development of DNS.

### Physical findings and laboratory results at first consultation

The results show that more severe consciousness disturbance at the time of first hospital consultation is associated with higher likelihood of developing DNS. There have been sporadic reports that consciousness disturbance [26] and prolonged loss of consciousness [10] involved in acute CO poisoning are risk factors for developing DNS. Acute CO poisoning resulting from attempted suicide is often combined with other means of suicide attempt, such as alcohol use or drug abuse, and these multiple factors may result in aggravation or prolongation of consciousness disturbance.

In the present study, all cases underwent head CT on the day of admission. Cases with low-density area in the globus pallidus were significantly more likely to develop DNS. CO produces parenchymal necrosis in fragile areas in the cerebral gray matter, particularly bilateral symmetric necrosis of the globus pallidus, which has been reported to be characteristic of CO poisoning [8]. Other areas often affected include the hippocampus, cerebellum and substantia nigra, where affected parts appear as low-density areas on CT. While CO gas concentration multiplied by duration of exposure is considered an important determinant of the severity of acute CO poisoning as mentioned above, in clinical settings it is often difficult to accurately find out how long the unconscious patient has been exposed to CO. However, a report has proposed a cut-off value of 570 min as a duration of exposure above which abnormal CT/MRI findings are predicted to be observed at the initial stage [27]. Using this threshold, it should be possible to estimate, from head CT findings at emergency admission, whether or not the patient has had prolonged exposure. A number of reports have identified abnormal CT/MRI findings [26] as a risk factor for developing DNS, which is also the case with this study. On the other hand, 20% of the cases with no abnormal CT findings did develop DNS, suggesting that even cases without abnormal findings require attention to the clinical course.

Hematology results show that abnormally high CK, CK-MB and LDH levels are significantly associated with the development of DNS. In acute CO poisoning cases, hypoxia and impaired cellular respiration caused by CO induce damage to multiple organs. These high CK levels are caused by damage to skeletal muscles. In this regard, the effect of pressure ulcer formation, which was seen in a number of cases due to prolonged immobility in the same position, should also be taken into account. LDH is an enzyme found in almost all cells and is released into the bloodstream when cells are damaged. As such, it is used as an indicator for assessing the severity of general condition [28]. The high LDH and CK-MB levels are considered to have been caused by myocardial injury. While these high levels have both been caused by prolonged exposure to CO, the hematological changes observed are regarded as nonspecific and not characteristic of CO poisoning [11].

The CO -Hb level only indicates the binding ratio between CO and Hb. As such, it decreases with time once CO inhalation is stopped, and decreases more efficiently as a result of oxygen administration in the ambulance. For this reason, CO-Hb levels following emergency admission are not directly associated with the degree of systemic tissue damage, as seen in the results of this study, which failed to show a significant association between CO-Hb levels and the development of DNS. In fact, the non-DNS-developing group had a higher mean CO-Hb level. However, a high CO-Hb level at admission indicates that the patient has been exposed to correspondingly high levels of CO, a fact which should be borne in mind in taking measures in the clinical setting.

Overall, F3 was the most common psychiatric main diagnosis according to ICD-10. In the psychiatric assessments of severity, no significant difference was observed between the DNS-developing and non-DNS-developing groups in BPRS score, which assesses the severity of psychiatric symptoms only, or in LCU score, which assesses the intensity of life events. However, the GAS score was significantly lower in the DNS-developing group. The GAS is a comprehensive functional assessment scale covering psychological, social and occupational functions, with lower GAS scores indicating more severe conditions. Assessment was conducted by a psychiatrist who collected, immediately after admission, information on the immediately preceding circumstances from the patient’s family, etc. In the DNS-developing group, patients’ overall function was significantly poorer. Clinically, the GAS score was independent of the development of DNS, and a lower GAS score was not considered to be a predictor for development of DNS. However, individuals with a lower GAS score may be profiled as individuals with worsening psychiatric symptoms to the extent that they affect physical and social functioning. These individuals may carefully plan to commit suicide by actions such as selecting a location away from public view, sealing up a car or room, and combining multiple methods of suicide. These actions would result in exposure to CO sufficient to subsequently cause DNS.

### Clinical course after hospitalization

Given that the maxim period before the onset of intermittent CO poisoning was 35 days in this study, at least 5 weeks’ follow-up is believed necessary. Length of hospital stay was inevitably longer in the DNS-developing group, since patients in this group needed time to recover from DNS. Similarly, the number of HBO therapy sessions was larger in the DNS-developing group, since approximately 60 HBO sessions are required once DNS develops.

On the other hand, 62% of all cases and 77% of the cases in the DNS-developing group received HBO therapy on the day of emergency admission. This suggests that, despite its efficacy in acute CO poisoning and DNS cases, HBO therapy may not be able to completely prevent the development of DNS even if administered during the initial stage of treatment.

## Conclusion

The profile of cases at high risk of developing DNS is expected to include a clinical picture consisting of: the patient’s selection of CO exposure as a means of suicide attempt in such serious mental condition as to affect his/her social and living functions; serious consciousness disturbance at admission due to acute CO poisoning, with a JCS score at or above 100; head CT findings indicating hypoxic encephalopathy; and abnormally high CK, CKMB and LDH levels detected by a blood test. In these cases, active consideration should be given to HBO therapy from an early stage after explaining to the patient’s family members the risk of developing DNS, and at least five weeks’ follow-up is believed necessary, during which due consideration should be given to the potential development of DNS.

The results of this study are tentative. Plans are to collect more substantiating data and conduct additional studies in the future.

## Competing interests

The authors declare that they have no competing interests.

## Authors’ contributions

Conception and design: KK, KO and AS. Acquisition of Data: KK, KS, NK, AK, JY, MYU, YY, AM, TM, YS and FK. Analysis and Interpretation of Data: KK, HK and KO. Drafting the manuscript: KK. Final Approval of the Completed Manuscript: AS. All authors read and approved the final manuscript.

## Pre-publication history

The pre-publication history for this paper can be accessed here:

http://www.biomedcentral.com/1471-227X/14/3/prepub
